# Gastrointestinal Stromal Tumors Mimicking Ovarian Mass: A Case Report

**DOI:** 10.7759/cureus.58320

**Published:** 2024-04-15

**Authors:** Anubha Dande, Sandhya Pajai, Neema Acharya, Ketav S Joshi, Dharmesh J Patel, Aishwarya Gupta

**Affiliations:** 1 Department of Obstetrics and Gynaecology, Jawaharlal Nehru Medical College, Datta Meghe Institute of Higher Education and Research DU, Wardha, IND

**Keywords:** mass in abdomen, tumor markers, histopathology, gastrointestinal stromal tumors, ovarian mass

## Abstract

Gastrointestinal stromal tumors (GIST) are common mesenchymal tumors of the gastrointestinal tract. Some somatic factors have been linked to an increased incidence risk. The diagnostic process for GIST poses difficulties since it bears limited resemblance to ovarian masses, given its manifestation through symptoms like abdominal pain, abdominal mass, fever, weight loss, and loss of appetite. Patients with GIST usually exhibit clinical symptoms and signs of an abdominal mass and chronic pelvic pain might look like an ovarian mass, and diagnosed as GIST on histological examination. A 50-year-old woman presented to the gynecology outpatient department with complaints of an abdominal lump accompanied by pain and decreased appetite persisting for five months, leading to a preliminary diagnosis of an ovarian mass. Further evaluation by histopathological examination was confirmed to be GIST on the final diagnosis.

## Introduction

Gastrointestinal stromal tumors (GIST) are a rare class of tumors, which can appear anywhere along the tubular gastrointestinal tract [1]. The majority of GISTs are observed to originate from the stomach with an incidence of 50%-60% followed by small bowel, large bowel, and esophagus with a reported incidence of 20%-30%, 10%, and 5%, respectively. The remaining 5% may have an origin elsewhere in the abdominal cavity in omentum and mesentery and shows the rarity of the case [2]. Numerous studies conducted in the late 1990s found similarities between the interstitial cells of Cajal and the neoplastic cells of GISTs [3-5]. They can be asymptomatic and are sometimes discovered as incidental findings during surgery or radiological tests. Abdominal mass, pain in the abdomen, and bleeding through the gastrointestinal tract are usual clinical symptoms observed in patients with GIST tumors [6]. Common clinical symptoms caused due to ovarian mass are weight loss, indigestion, bloating, acid reflux, abdominal pain or distension, constipation, dyspnea, and vaginal bleeding. Patients might have a feeling of bulge in their abdomen. Ovarian cancer is discovered late in the clinical course of the disease due to a lack of early symptoms and screening procedures [7]. As clinical presentations of an ovarian mass and GISTs are nearly similar, it is challenging for gynecologists to diagnose GISTs pre-operatively, and a biopsy study is suggested before final diagnosis is concluded.

## Case presentation

This is a case of a 50-year-old female with an obstetric score of P3L3A0, who visited the outpatient department of our tertiary care hospital. Major complaints were pain in the abdomen for four to five months, which originated in the left iliac region and radiated toward the back. The pain was on and off throbbing type in nature and temporarily relieved by medical management given by private practitioners. The feeling of a mass in the abdomen, which was located in the left iliac and left hypogastric region. The mass was fixed with no tenderness. This mass progressively increased in size from 5 cm x 6 cm to the current size of 10 cm x 12 cm on ultrasonography evaluation. The initial scan was done three years ago, as the patient had complained of pain in the abdomen. It suggested a mass of 5 cm x 6 cm size but the location was not specified. Along with these symptoms, she had bowel and bladder irregularities like increased frequency of micturition, and urinary hesitancy, and along with difficulty in passing stools. The patient had a history of loss of appetite and weight for three months. The patient did not have any other significant co-morbidities like hypertension, diabetes, hypo/hyperthyroidism, tuberculosis, any major operative procedure, or a history of similar complaints in the past. On physical examination, the patient was vitally stable with no supraclavicular lymphadenopathy. Per-abdomen examination revealed a soft abdomen, non-tender, fixed solid mass of about 10 cm x 12 cm in the left iliac region with an irregular surface and hard consistency. No ascites were observed, and transillumination in the mass was absent. The patient was subjected to basic lab investigations along with tumor markers, which were found to be within normal limits as mentioned in Table 1 apart from LDH values, which were found to be higher.

**Table 1:** Laboratory profile of the patient AFP: alpha fetoprotein; HCG: human chorionic gonadotropin; CA19-9: protein cancer antigen 19-9; LDH: lactate dehydrogenase; CA125: protein cancer antigen 125

Tumor marker	Observed value	Normal range
AFP	0.946 IU/mL	0-40 IU/mL
Beta-HCG	2.39 mIU/mL	<5 mIU/mL
CA19-9	15.8 U/mL	<37 U/mL
CA125	22 U/mL	<35 U/mL
LDH	572 U/L	120-246 U/L

**Figure 1:**
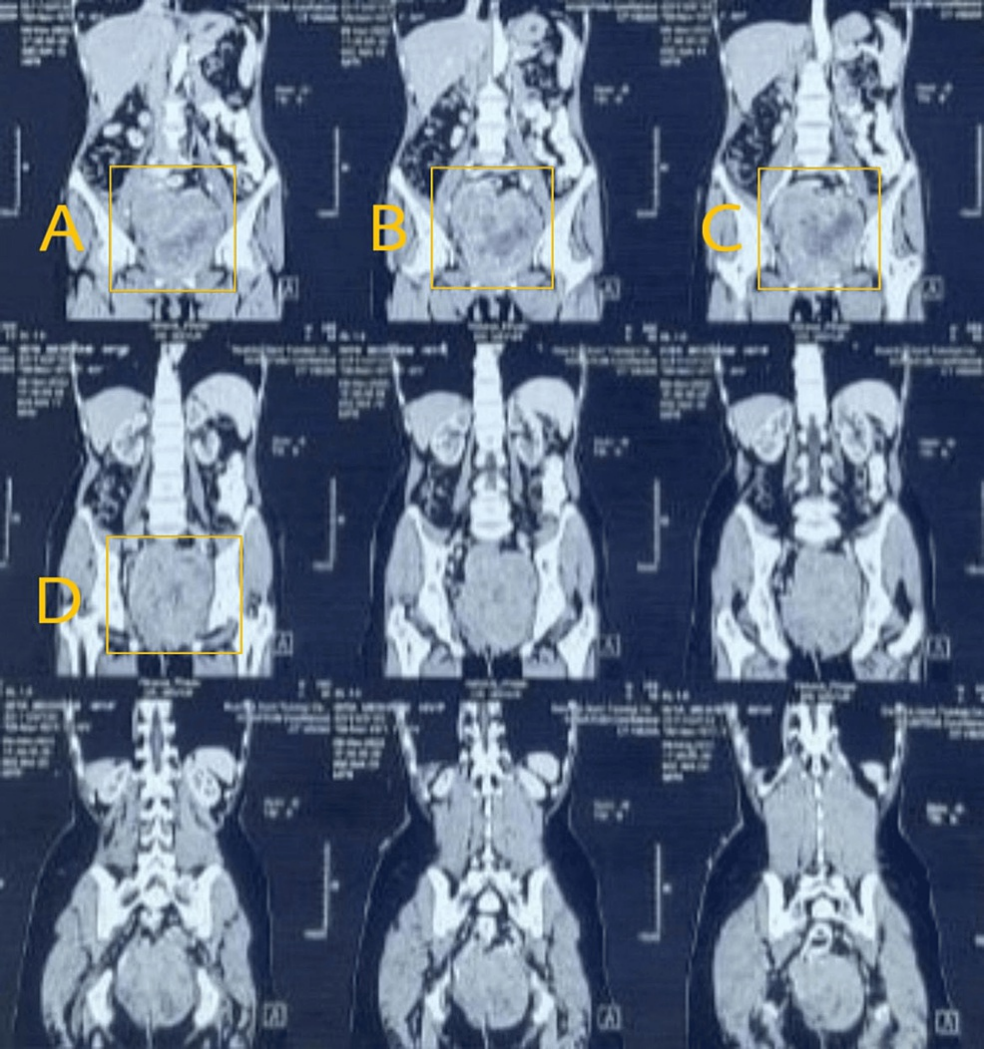
MRI showing GIST Highlighted areas of A-D showing the tumors in different panes GIST: gastrointestinal stromal tumors; MRI: magnetic resonance imaging

**Figure 2:**
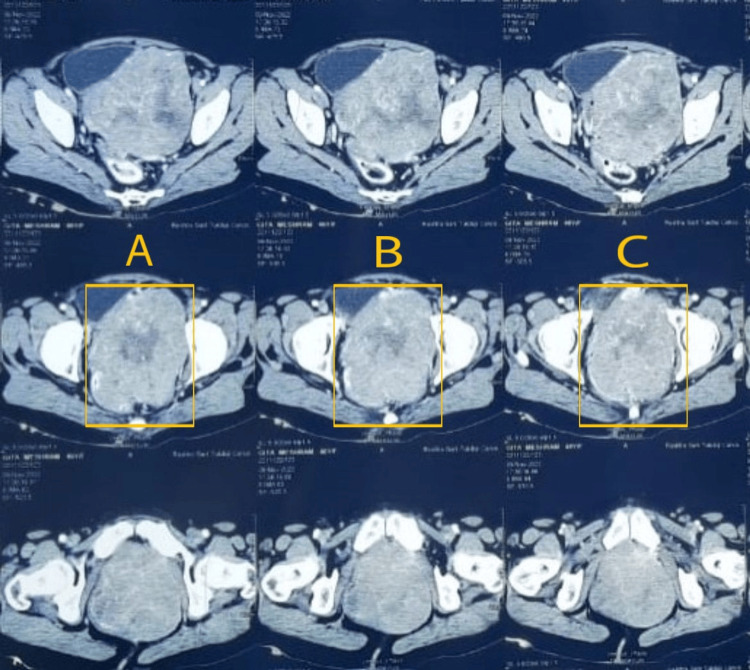
An enhanced MRI image showing a large, well-defined soft tissue mass with heterogenous enhancement in a bulky tumor The area highlighted in A-C shows the tumor MRI: magnetic resonance imaging

The sonography report was suggestive of an ill-defined 10.2 cm x 3.5 cm x 15.2 cm hypoechoic lesion with a volume of 117 mL, with internal vascularity located in the left side of the pelvis involving the lateral wall of the cervix, vagina, anterior wall of the rectum with a possibility of neoplastic origin; the ovaries were not separately visualized. The left side of the pelvis had a large, heterogeneously enhancing, lobulated mass lesion with several calcification foci as noted in contrast-enhanced computed tomography (CECT) abdomen. The uterus was laterally shifted to the right, but the fundus and body were found to be normal. Multiple artery channels were visible within the lesion, which was about 14 cm x 13 cm x 13 cm in size, and had prominent enhancement in the left ischiorectal and peri-urethral regions (Figure 1-2).

**Figure 3:**
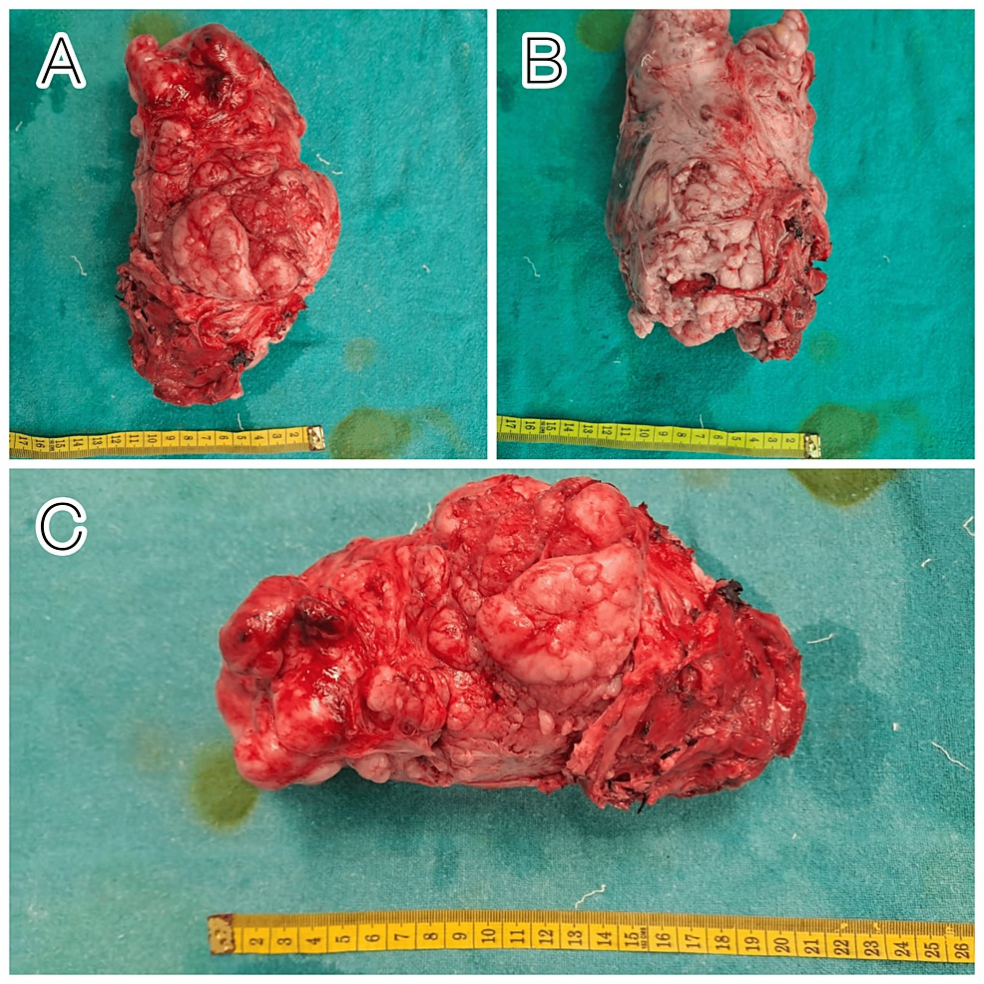
Excised specimen from different planes; solid mass removed after extensive dissection with no clear demarcation between the uterus, ovaries, and gastric organs A and B-vertical planes, C-horizontal plane

CECT findings were suggestive of multiple feeding arteries from the internal iliac vessels. Even after several examinations, the cause and origin of the tumor remained elusive. Consequently, both ureters were stented with double-J catheters, and bilateral uterine artery embolization was performed to halt the blood supply to the mass before proceeding with exploratory laparotomy. The biopsy report of the mass revealed a benign mesenchymal tumor. On exploratory laparotomy, dense anterior and posterior adhesions were observed between the mass, bladder, rectum, and genital organs. Since the uterus and ovaries were not separable from the mass, hysterectomy with bilateral salpingo-oophorectomy was performed with extensive dissection of anterior and posterior surgical planes. The anterior wall of the rectum was involved and excised along with the mass, and a colostomy was performed (Figure 3).

**Figure 4:**
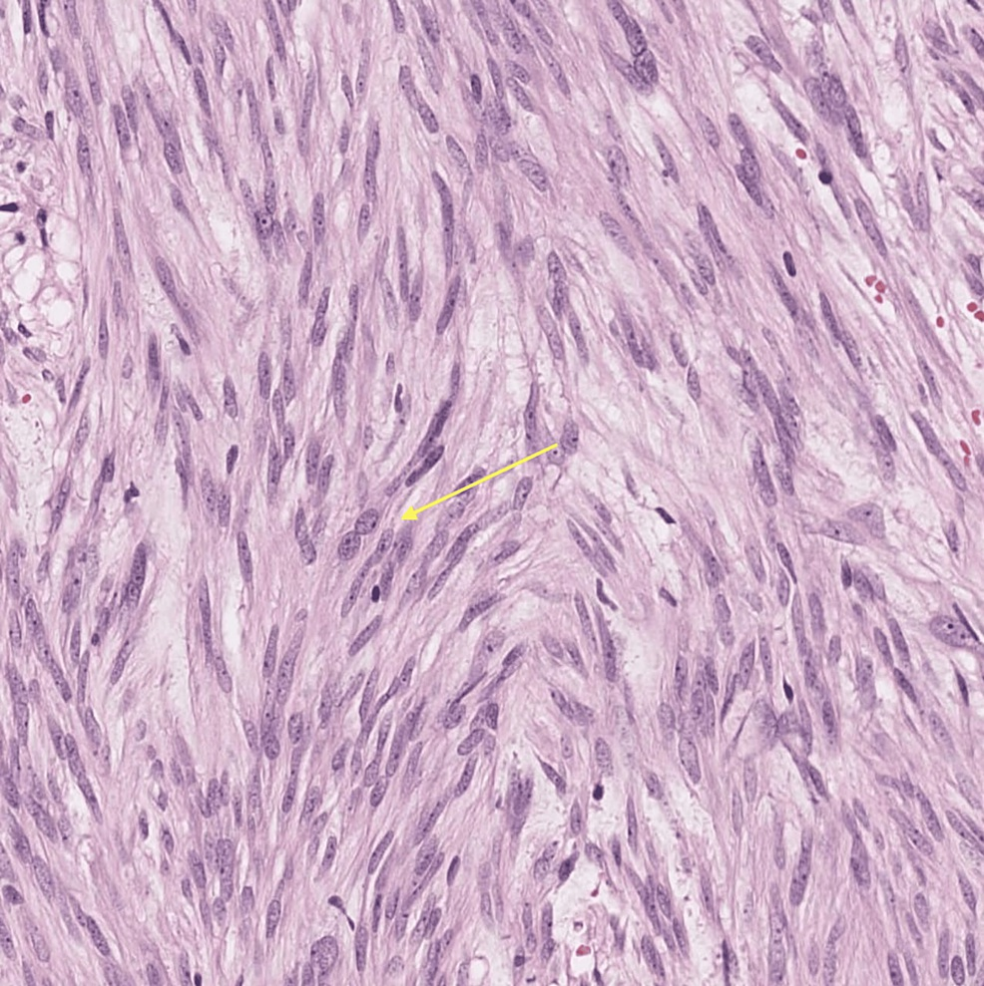
Histopathological analysis (40x magnification) of the excised specimen indicative of a GIST The yellow arrow indicates the spindle and epithelioid cells with abundant eosinophilic cytoplasm, a common feature of GIST GIST: gastrointestinal stromal tumors

The abdomen was closed in layers with peritoneal drains in place. The histopathological features of the tissue were suggestive of GIST (Figure 4). 

It was found to be positive for CD117, which is a proto-oncogene activated in GIST. Apart from that it was also positive for CD34 and vimentin. The mitotic count was ≤5/50 HPF. She was managed in the surgical intensive care unit with higher antibiotics and multiple blood transfusions in a 15-day post-operative period and posted for adjuvant chemotherapy.

## Discussion

GISTs are rare cancer that can be clinically arduous to diagnose in female patients having abdominal and pelvic masses, clinically presented as the presence of a tumor or per vaginal bleeding [8,9]. Occasionally, these tumors can cause pelvic masses, which may be misdiagnosed as gynecologic issues. Tumors originating from the distal small intestine, sigmoid colon, or rectum could be mistakenly identified as gynecologic neoplasms. Mutations in genes regulating cell growth and cell division such as platelet-derived growth factor receptor alpha (PDGFRA) and KIT are reported to be associated with the majority of GISTs, leading to the formation of tumors by uncontrolled cell proliferation [10,11]. Recto-sigmoid GIST and small bowel tumors present as a difficult, heterogeneous pelvic tumor. The clinical and imaging features of GIST can mimic an ovarian tumor as it neither has any particular symptoms nor any imaging features clearly linked with it. This case also presented similar complaints to those in ovarian tumors. Even, the radiological diagnostic modalities were inconclusive of the associated pathology. Histopathological analysis was used for a conclusive definite diagnosis. There is no research evidence showing GISTs to have any characteristic symptoms or associated radio imaging specifications; most of the clinical symptoms and imaging features match those of an ovarian tumor. CECT is the most preferred diagnostic imaging method. When segmental resection is not possible, a broad, en bloc resection must be performed [12]. A variety of tumor markers and imaging modalities can play a crucial role in differentiating the diagnosis between ovarian mass and GISTs with final confirmation by histopathological examination. Findings on gross histopathological examination frequently include mushy, fish-flesh-like appearances. Necrotic tumors and cystic degeneration are both possible. Spindle cells makeup about 70% of the total tumor subtype population, 20% of epithelioid cells, and 10% of mixed phenotypic cells. GISTs express h-caldesmon (60%), smooth muscle actin (30%), CD34 (70% of tumors), and S-100 (5% of tumors) as other markers. Another novel marker DOG1 was found in GIST, which is a piece of cDNA that codes for an unidentified protein. When a GIST is negative for c-Kit, this marker may be useful, attributed to its high sensitivity in the detection of GISTs (94.4%) and is positive in 80%-90% of GISTs [13,14]. Due to their notable positivity for the CD117 marker, which is a result of the c-kit gene, these tumors distinguish themselves from other mesenchymal tumors of the gastrointestinal tract, such as smooth muscle tumors (leiomyoma and leiomyosarcoma) or those with Schwann cell differentiation (neurinoma). The preferred approach for localized GISTs involves complete surgical removal followed by treatment with tyrosine kinase inhibitors [14]. Pre-operative GIST diagnosis is still rare because of the variety of ways these tumors present themselves and the lack of distinctive features on imaging testing. It is advised that gynecologists understand extra-ovarian pathology in circumstances where a patient presents with an atypical pelvic tumor. 

## Conclusions

It is crucial for clinicians to be cautious in cases of pelvic masses and rare entities like GISTs be taken into account. Prompt identification and precise diagnosis of GISTs are essential for optimizing patient care with a multidisciplinary approach from gynecologists, general surgeons, radiologists, and pathologists, which can result in a good prognosis. Timely intervention, including surgical resection and targeted therapy, can significantly improve the outcome and quality of life for patients with GISTs. Further research can enhance the insights gleaned from advanced diagnostic imaging modalities like MRI, aiding in the differentiation of various abdominal masses and facilitating meticulous pre-operative strategizing.
